# Remarks on the Treatment of Fractures*Delivered at the Third Annual Meeting of the American Academy of Railway Surgeons, held at Chicago, Ills., Sept. 23,24, 25, 1896.

**Published:** 1897-08

**Authors:** R. Ortega

**Affiliations:** Chief Surgeon to the Mexican International Railway, Ciudad Porfirio Diaz, Mex.


### Remarks on the Treatment of Fractures.*

BY R. ORTEGA, M.D.

Chief Surgeon to the Mexican International Railway, Ciudad Porfirio Diaz, Mex.

I want to talk about a treatment or practice which gives the patient entire relief at once from pain and puts him in good condition for work as soon as the fracture is solid. As soon as a patient breaks a bone, I make massage from the lower part of the fractured limb to the upper part; we commence the massage very gently just on the skin, with anything, oil, or soap and water, salve or vaselin to allow the hand to rub without hurting. Ten or fifteen minutes afterward we do the massage on the muscles, always from the end of the member up, never making pressure; in ten or fifteen minutes after the pain is gone and we can reduce the fracture without chloroform or pain. As soon as the fracture is reduced we roll it in a simple flannel bandage, from the end of the member upward; if it is in the arm we put the arm in a simple handkerchief from the neck; if it is in the upper arm, the weight of the arm is enough to keep the fracture in good position. And the next day we do the same treatment—a little massage and a little movement in all the joints of the broken limb. I have done this treatment for a year. I have had but few cases, as my practice is very small. One boy about eighteen years old, had a wheel from which he fell and broke his right arm in the middle with a compound fracture, the upper end of the humerus projecting through the skin. Dr. Lord reduced this fracture, put on a good dressing and an apparatus, and he called the next day to see the patient; the patient wanted to cross the river. I saw the boy with Dr. Lord; we took off the apparatus, the wound was in good condition, aseptic because the doctor had put a very nice and good dressing on. I told him I was going to take off the apparatus as soon as I brought the boy over the river. I did so and then applied a bandage, and made motion in the elbow and other joints, and in fifteen days this boy was at

*Delivered at the Third Annual Meeting of the American Academy of Railway Surgeons, held at Chicago, Ills., Sept. 23, 24, 25, 1896.

work as before the injury. There was a compound fracture of the humerus and simple fracture of the ulna, and in fifteen days later the boy recovered and worked as before, all the bones completely healed. I had one day two cases; one a small boy, about six years old, broke the humerus of the right arm, not a compound fracture; I applied massage at once, and in ten days this little boy climbed trees and did almost everything. The same day in the shop, a workman broke the humerus in the middle, and the fracture was displaced; I did massage and put the fracture in position and rolled in a bandage, and I left this arm without an operation; I did massage every day and about twenty days afterward this patient was in good condition. I do not remember the details of other cases, but I want to call your attention to this treatment. It gives a little more trouble to the doctor, but that does not count; the patient does not suffer and is able to work very soon. My company does not give wages to people when they are sick, and I think this treatment is very good because it does not keep patients two or three months without working; they can go to work in fifteen or twenty days.—*The Journal Am. Med. Ass'n.*

